# A Dynamic Calibration of Optical Fiber DTS Measurements Using PEST and Reference Thermometers

**DOI:** 10.3390/s22103890

**Published:** 2022-05-20

**Authors:** Yaser Ghafoori, Andrej Vidmar, Andrej Kryžanowski

**Affiliations:** Faculty of Civil and Geodetic Engineering, University of Ljubljana, Jamova Cesta 2, 1000 Ljubljana, Slovenia; andrej.vidmar@fgg.uni-lj.si (A.V.); andrej.kryzanowski@fgg.uni-lj.si (A.K.)

**Keywords:** optical fiber DTS, calibration, PEST, temperature, thermometer, Raman scattering

## Abstract

Temperature measurements are widely used in structural health monitoring. Optical fiber distributed temperature sensors (DTS) are developed, based on Raman spectroscopy, to measure temperature with relatively high accuracy and short temporal and spatial resolutions. DTS systems provide an extensive number of temperature measurements along the entire length of an optical fiber that can be extended to tens of kilometers. The efficiency of the temperature measurement strongly depends on the calibration of the DTS data. Although DTS systems internally calibrate the data, manual calibration techniques were developed to achieve more accurate results. Manual calibration employs reference sections or points with known temperatures and the DTS scattering data to estimate the calibration parameters and calculate temperature along the optical fiber. In some applications, manual calibration is subjected to some shortages, based on the proposed fiber installation configuration and continuity of calibration. In this article, the manual calibration approach was developed using the model-independent Parameters Estimation (PEST), together with the external temperature sensors as references for the DTS system. The proposed method improved manual calibration in terms of installation configuration, continuity of dynamic calibration, and estimation of the calibration parameters.

## 1. Introduction

The optical time domain reflectometry (OTDR) technique is employed in optical fiber distributed temperature sensors (DTS) for temperature measurements. Short pulses of light are launched by the system’s laser into the optical fiber core. Small numbers of Raman backscatter lights (Stokes and anti-Stokes) are generated due to the vibrational energy transition between the photons and molecules of the cable’s core. The travel time of light is measured by the system, and the generated scattered photons are detected by the fast photonic detector at regular time intervals [1]. The optical fiber DTS is equipped with an optical spectrum analyzer and the required software to resolve different wavelength peaks (Stokes and anti-Stokes), calculate the temperature, and calculate the distance between the measuring point and the system using the detected backscattered photons. The intensity of anti-Stokes scattered photons is influenced by the temperature variation; therefore, the intensity ratio of anti-Stokes to Stokes scattered photons $\left(\frac{P_{as}}{P_{s}}\right)$ is used to measure the absolute temperature $\left(T\right)$ along the optical fiber [2,3].

(1) $$\frac{1}{T}=\frac{1}{T_{r}}-\left(-\frac{K_{B}}{h_{p}c_{l}\nu_{n}}\right)\ln[\frac{R\left(T\right)}{R\left(T_{r}\right)}]$$

where $T_{r}$ [K] is the known temperature of the reference section, $R\left(T_{r}\right)$ and $R\left(T\right)$ are the intensity ratio of anti-Stokes to Stokes scattered photons for known and unknown sections, respectively, $h_{p}$ $\left[\frac{m^{2}kg}{s}\right]$ is Planck’s constant, $c_{l}$ $\left[\frac{m}{s}\right]$ is the light velocity, $K_{B}$ $\left[\frac{m^{2}kg}{s^{2}K}\right]$ is the Boltzmann constant, and $\nu_{n}$ [m] is the difference in wavelength between the incident and scattered photons. 

The intensity ratio of backscattered photons and, consequently, the accuracy of the temperature measurement are related to the number of detected Stokes/anti-Stokes photons. The accuracy of the temperature measurement is also strongly influenced by the power and efficiency of the laser. The efficiency of a laser is inversely proportional to the fourth power of its wavelength [4,5]. However, selecting a very short wavelength will lead to electrical energy transition, instead of vibrational energy transition. The optical power of the emitted light decreases with increasing distance from the laser source. This steady loss of power with the lengthening of the cable is called the differential power loss. Therefore, a smaller number of backscattered photons will be detected by the system from a far distance along the cable [6,7]. The accuracy of the temperature measurement can be improved by increasing the integrated time of the system, which gives the system a long time for the collection of backscattered photons. However, in some applications, it is a requirement to provide measurements in short time intervals to detect any fast adverse changes in the structure. The cable’s attenuation can also be due to local adverse effects, such as bending or the presence of splicers and connections. Additionally, the DTS system instruments are temperature sensitive, affecting the temperature measurement process. 

The DTS data are automatically calibrated by the system using an internal mechanism to eliminate the attenuation effect. Typically, the longitudinal attenuation is corrected by introducing an expected differential loss of power along the optical fiber to the DTS system. Most of the standard optical fibers used for environmental DTS applications have losses of around 0.3 dB/km that can be assigned in the DTS system for calibration [8]. Within the DTS system (commonly between the directional coupler and sensing fiber), a reference coil of fiber with a typical length of 50 m to 100 m is placed to correct the attenuation effects related to the DTS instrumentations [9,10]. The instrumental error is corrected by continuous temperature measurements of the internal reference coil with a precise internal thermometer, and these measurements are compared to the temperature obtained from the scattering analysis. Internal calibration can be improved by assigning a reference section or point with a known temperature along the fiber [8] and placing the precise thermometer (e.g., PT100), which is attached to the DTS system [11], at this reference point. 

In a stationary condition, a high-accuracy temperature measurement is obtained by the internal calibration of the DTS system; however, the accuracy of the DTS internally calibrated temperature is dramatically reduced when rapid temperature changes occur along the fiber. Additionally, the accuracy of the temperature measurement is decreased when short integration time and sample intervals are employed. Internal calibration can also be subjected to errors due to the assignment of a constant differential loss over the entire cable length. Therefore, manual calibration of the DTS data is required in many practical applications for high-accuracy temperature measurement. In manual calibration, the scattering data obtained from the DTS system are employed together with the temperature measurements in reference sections/points to calculate the calibration parameters. In other words, the role of the internal software is partially eliminated by manual calibration, and the temperature is calculated using the obtained intensity of Stokes and anti-Stokes backscattered photons, and by considering the different interference factors. The manual calibration technique was developed based on the equation introduced by Farahani and Gogolla in 1999 [12]. The temperature along the cable at a distance of $z\left[m\right]$ from the laser source of the DTS system is calculated from the detected power of Stokes $P_{s\left(z\right)}$, anti-Stokes $P_{as\left(z\right)}$, and the calibrated parameters.

(2) $$T\left(z\right)=\frac{\gamma }{ln\frac{P_{s\left(z\right)}}{P_{as\left(z\right)}}+C-∆\alpha z}$$

where $T\left(z\right)$ is the temperature [K] at distance z [*m*], γ [K] is a calibration parameter related to the energy shift between the incident photon and the Raman scattered photon, *C* is a dimensionless calibration parameter that represents the influences of the incident laser properties and the DTS instrument itself, and $∆\alpha =\alpha_{s}-\alpha_{as}$ $\left[m^{-1}\right]$ is the differential attenuation of the backscattered Stokes and anti-Stokes signals [13]. γ is dependent on the distribution of quantum states and can be estimated by Equation (3) [13,14]:

(3) $$\gamma \approx \frac{h_{p}\Omega }{K_{B}}$$

where $\Omega =\omega_{0}-\omega_{s}$ is the difference in frequency between the incident laser pulse and the Stokes backscattered photon. 

**Figure 1:**
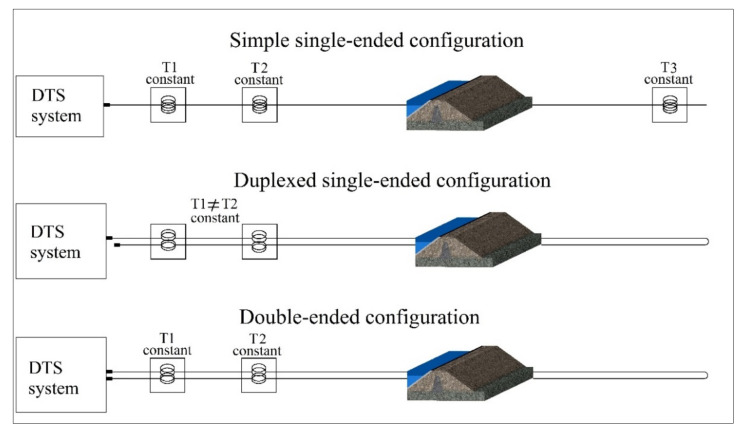
Optical fiber installation configurations for manual calibration purpose.

The three calibration parameters (γ, *C*, and ∆α) are independent. In manual calibration, the calibration parameters are obtained by comparing the temperatures measured in reference sections/points with thermometers and the temperature calculated from the scattering data reported from those sections/points. The manual calibration can be performed statically. The calibration parameters are obtained from an initial measurement integration time, which is assumed to be uniform along the cable and constant over time [9]. However, in many applications, the calibration parameters should be determined for each measurement time step, called dynamic calibration. The calibration is generally performed in three configurations: simple single-ended, duplexed single-ended, and double-ended measurements [15]. In a simple single-ended configuration, the temperature is only measured from one end of the cable connected to the DTS system. In the duplexed single-ended configuration, the cable is formed from two co-located fibers following the same path. The cable is connected to the DTS system from one side. However, the system provides two temperature measurements for each point along the optical cable; one from the DTS outward and the other from the cable coming back to the system. In the double-ended configuration, the temperature measurement is performed from both ends of the fiber connected to the DTS system [10]. These three installation configurations are shown in Figure 1.

The three calibration parameters in Equation (2) can be calculated based on the installation configuration, using different approaches. If three reference sections or reference points are presented, the three parameters can be calculated by simultaneously solving Equation (2) for the reference sections [10]. The calibration can also be performed using two reference sections or points in the absence of three references. If one of the reference sections is long enough, the differential power loss (∆*α*) is calculated from the Stokes/anti-Stokes intensity of the two ends of the section using Beer’s law. ∆α can also be estimated using two reference sections/points with the same temperature. Obtaining the value of ∆α, both references can be used to calculate the other two calibration parameters. 

In practice, for dynamic calibration, the reference section of the fiber is immersed within a bath of water with a constant temperature, while its temperature is measured continuously by a separate temperature sensor [7,8,14,16]. The main limitation of this approach is the complexity of forming the reference bath and keeping its temperature constant over time. The temperature distribution within a reference bath can also be non-homogenous. In some applications, water circulation and mixing techniques were used to equalize the water temperature within the reference bath [13,17]. Additionally, providing a reference section at the end of the cable to improve the calibration is not applicable in some field applications. A comparative overview of different calibration techniques and their potential disadvantages can be found in [15]. For the most conventional calibration techniques, the calibration parameters are found by minimizing the root mean square error (RMSE) between the calibrated temperatures obtained from the reported Raman spectra and the observed temperatures in the reference baths [10,18,19].

The main objective of this work is to improve the manual calibration of DST measurements in terms of the installation configuration and calibration process. Nowadays, real-time thermometers are available on the market to provide accurate temperature measurements remotely. This article proposes an installation configuration where the real-time temperature sensors are wrapped up by the optical fiber to form the reference sections. To obtain the calibration parameter, a calibration process was developed using the model-independent Parameters Estimation (PEST) tools. PEST is a software package developed by John Doherty for the calibration and uncertainty analysis of complex environmental models [20]. The main advantage of the PEST application is that it performs high-accuracy parameters estimation in a relatively short computing time, even for a very complex calibration model. In addition to the uncertainty analysis, the calibration model’s sensitivity to each parameter can also be calculated by PEST. The calibration method developed in this article eliminates the need for a reference bath, and increases the calibration capability in terms of the accuracy, continuity, and simplicity of installation and operation.

## 2. Materials and Methods

### 2.1. Installation Configuration

**Figure 2:**
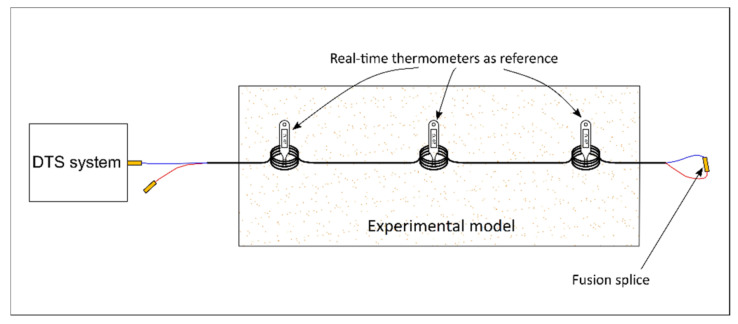
Installation of optical fiber and temperature sensors as references.

An optical fiber DTS system (manufactured by Silixa Ltd. [11]) was employed to measure the temperature within a laboratory model that was designed to study seepage propagation in sand. In addition to the temperature measurement, the Raman scattering data (Stokes and anti-Stokes intensities) provided by the DTS system were used in the calibration process. A duplexed single-ended configuration was performed. Temperature sensors were embedded within the sand to calibrate the DTS data. The temperature sensors can be installed in the center of sensory rings formed by the optical fiber, or the fiber can be wrapped around the sensors to create the reference sections. The scheme of installation configuration for the calibration purpose is shown in Figure 2. Temperature sensors can be installed in any proper location along the optical fiber to form the reference section for calibration, including within the monitored structure. 

**Figure 3:**
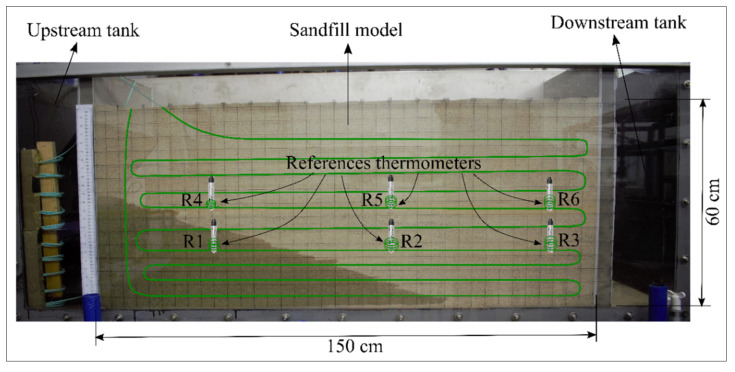
Scheme of temperature sensors and optical fiber arrangement within the sand.

The laboratory model consisted of a sand-filled box with dimensions of 150 cm × 50 cm × 60 cm (length, width, and height) together with the upstream and downstream tanks to study water propagation through the sand. A total of 130 m of optical fiber was embedded within the sand in vertical and horizontal layers to monitor the temperature variation due to water flow. A sampling interval of 25.4 cm (10 inches) was selected for temperature measurement, providing 520 reported temperatures within the sand for each integration time. Duplexed single-ended measurement was performed using an optical cable formed by two co-located 50/125 multimode fibers. A fixed value of 0.255 dB/km was assigned for the internal calibration of the DTS system, as suggested by the manufacturer [11]. Six temperature sensors (HOBO water level and temperature logger) were placed within the sand at two elevations (see Figure 3). HOBO sensors provide relatively accurate temperature measurements with a maximum error of ±0.44 °C [21]. The reference sections were formed by creating sensory rings around the temperature sensors using a 50.8 cm cable length. Considering the sampling interval, three temperatures were reported by the DTS for each sensory ring. Since the temperature of the first and third sampling intervals can be influenced by the adjacent intervals, the temperature of the middle sampling interval was considered for the calibration. The temperature of each reference point was measured by the HOBO temperature sensor every 60 s and by the optical fiber DTS at each 30 s time interval. Considering the duplexed measurement, 12 temperatures were reported by the DTS system for the six references. The response times for both the temperature sensors and the optical fiber were considered in the calibration process. The response time of the DTS system was determined as 60 s by a laboratory measurement [22], while the response time of temperature sensors was 5 min [21]. The real-time clocks of the DTS system and temperature sensors were precisely synchronized before the preparation of the model. 

### 2.2. Calibration by PEST

**Figure 4:**
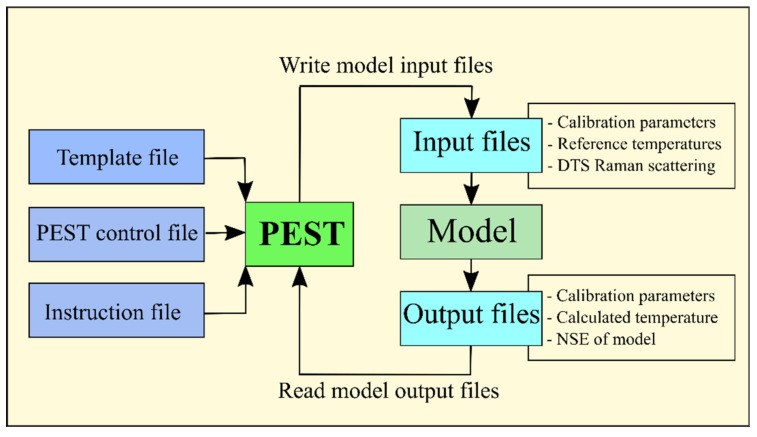
Schematic methodology of the PEST calibration.

The calibration parameters were estimated using the PEST tool. PEST is a software package used for calibration purposes, especially in the field of environmental modeling [23,24,25,26,27]. Calibration tasks, including uncertainty and sensitivity analysis, are performed automatically by the PEST software package. Specific tasks should be accomplished in PEST by the user. The adjustable calibration parameters, and their initial and boundary values, should be determined. Additionally, the PEST file preparation, operation, and result interpretation should be performed by the user [20,27]. Therefore, knowledge of the model and PEST file preparation is required for calibration tasks. Three files are needed to introduce inputs into the model for the PEST operation. The first is the template file introducing the variable parameters that control the calibration model. The second file is the instruction file assigning the values that PEST should read from the output file of the previous run. The third input file is the control file introducing all required characteristics of the problem and calibration model. Some mandatory sections should be provided in the control file. The problem dimension, the mode of PEST operation, and the termination criteria should be determined in the control data section. The variables that control derivative calculation are assigned in parameter groups. The parameters’ initial values, boundaries, and scale are set in the parameter data section. The name of the observation group, or the name of the file containing them, is provided in the observation group section, while the measured values of observation are assigned in the observation data section. The control file is completed by the model command line and model input/output sections [20,28]. The schematic methodology of the PEST application for calibration purposes is shown in Figure 4.

The calibrated model was prepared using Equation (2). The input data were assigned temperature measurements by the reference thermometers, and their respective scattering data (Stokes and anti-Stokes intensities), were obtained from the DTS system. The calibration parameters for each time increment were obtained using 12 temperature measurements in 6 different reference points. Using the obtained calibration parameters, the temperature along the entire length of the optical fiber was calibrated in each time increment for more than two hours of the experiment. 

## 3. Results and Discussion 

**Figure 5:**
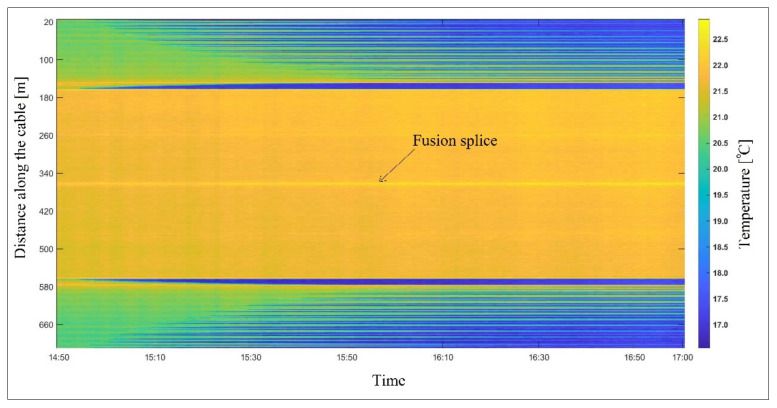
DTS temperature measurement during the seepage experiment.

The temperature measurement was conducted by the optical fiber DTS using the duplexed single-ended installation configuration in a sand model subjected to seepage flow. With seepage propagation in the model, the sand temperature decreased due to heat transfer by water. The measured temperatures by the DTS system (before manual calibration) are shown in Figure 5 for two hours of the experiment. 

**Figure 6:**
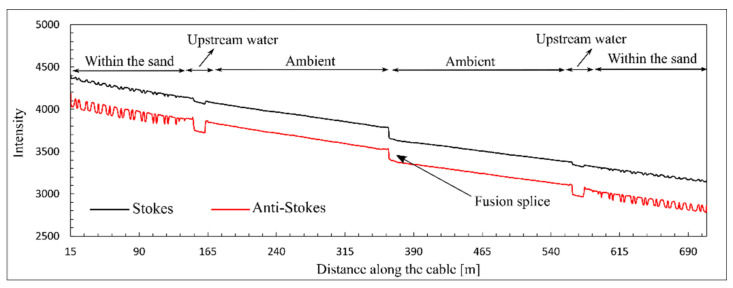
Stokes and anti-Stokes scattering at 15:50.

The temperature within the sand varied between 22 °C for initially dry sand and 16.5 °C when fully saturated. The temperature outside of the experimental model (ambient temperature) was constant during the measurement. The DTS temperature was calibrated using the proposed technique with PEST calibration tools for a time increment of 1 min. The scattering data and thermometers’ temperature measurements in the reference points were used to determine the calibration parameters. The Stokes and anti-Stokes intensities were provided by the DTS system. In Figure 6, the scattering data at 15:50 are presented. 

**Figure 7:**
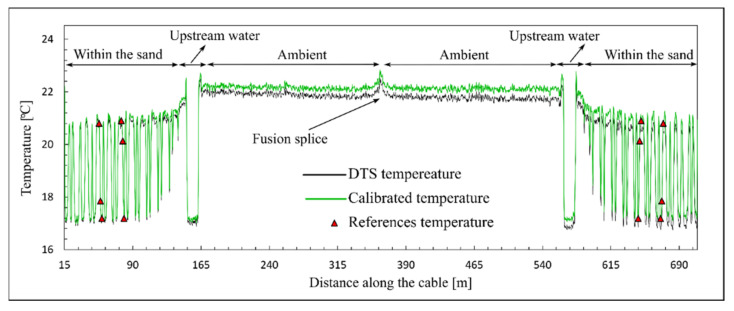
DTS measured temperature and calibrated temperature at 15:50.

As shown in Figure 6, the intensity of Raman scattering is decreased gradually at a constant rate due to differential laser power loss. Additionally, an abrupt drop in scattering intensity can be observed at 362 m from the DTS system. This is the loss of optical power associated with the presence of the fusion splice, where the two optical fibers have been fused together. Typically, there is a loss of 0.1 to 0.3 dB for each optical splice [29]. The splice loss shown in Figure 6 can be estimated from the intensities of Raman scattering reported ahead and behind the splice’s location. A power loss of about 0.1 dB was estimated for the fusion splice. This power loss can be considered as a local source of attenuation in the optical fiber. This drop in optical power is translated into the temperature measurement by the DTS system. The influence of this drop on the temperature measurement can be observed in Figure 5 for the entire measurement period. A higher temperature can be observed at this connection during the experiment. In Figure 7, the DTS reported temperatures and calibrated temperatures associated with the Raman scattering in Figure 6 are presented. 

**Figure 8:**
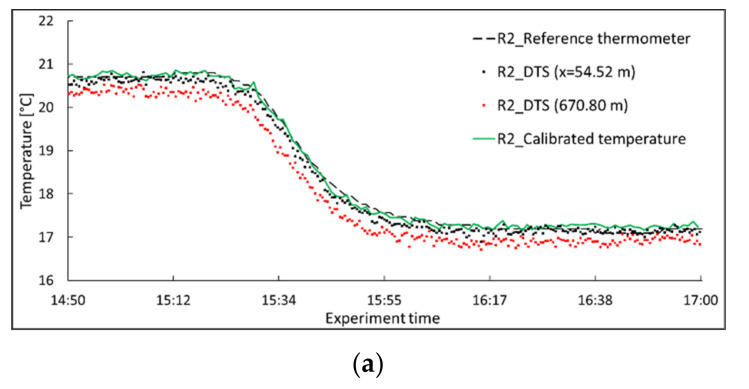

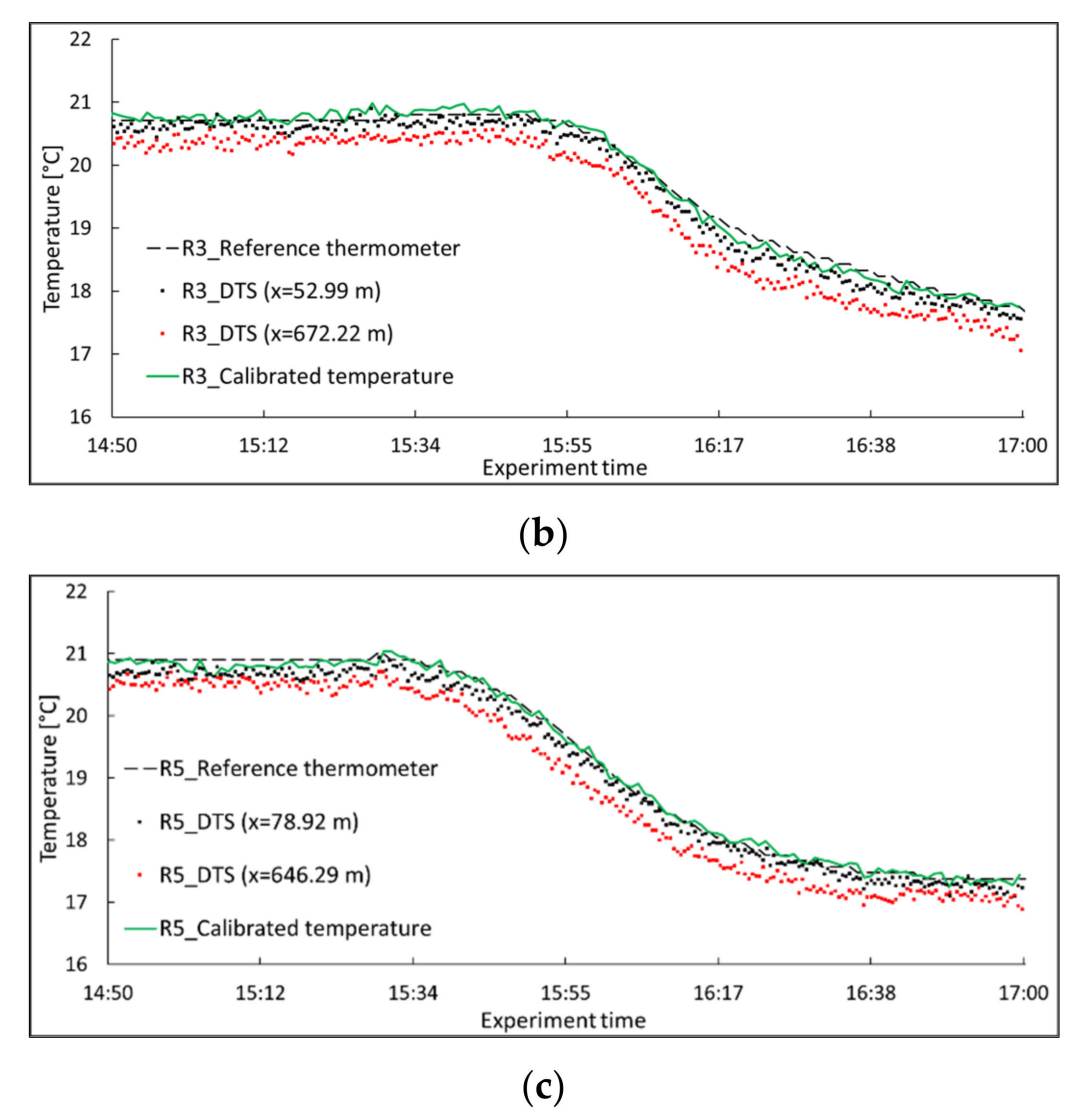
Temperature data for three reference points during the seepage experiment. (**a**) reference point 2, (**b**) reference point 3, and (**c**) reference point 5.

The influence of local power loss (the optical splice) on both the calibrated and the DTS measured temperatures was observed as a temperature jump at the splice’s location. The true temperature at the splice is the same as the ambient temperature. The effect of such local attenuation on temperature measurements can be significant. In a field measurement, the local attenuation can be due to optical connections and the presence of adverse effects, such as bending. As can be observed for the DTS temperature (black line in Figure 7), the abrupt attenuation in splice fusion not only affects the temperature measurement at the connection, but it also affects the temperature measurements beyond the connection. The temperature measurement accuracy beyond the optical connection is lower than the measurement accuracy close to the system. Adding the splice attenuation to the differential attenuation, the number of backscattered photons and, consequently, the accuracy of the DTS for temperature measurements decreased beyond the optical connection. The influence of the optical connection and differential power loss is shown in Figure 8a–c in a more explicit way for three reference points. Two DTS reported temperatures are presented for each reference point; one close to the DTS system and before the optical splice, and the other far from the system and after the optical splice. In Figure 8, the temperatures measured by the reference thermometers and the DTS, as well as the calibrated temperatures, are presented. 

**Figure 9:**
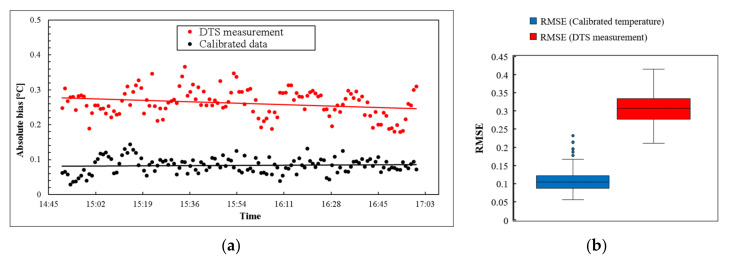
(**a**) Absolute bias for DTS and calibrated temperature compared to reference thermometers; (**b**) RMSE of DTS temperature and calibrated temperature.

As can be observed, the calibrated temperature is well-attuned to the temperature from the reference thermometers. The DTS temperature located far from the system reported a more significant error, due to differential power loss and the local attenuation in the splice fusion. The calibrated temperatures were obtained by the reported Raman scattering at both close and far distances from the DTS system. The effect of step attenuation on temperature measurements beyond the connection was eliminated by the performed calibration technique. It can be observed that the proposed calibration has the ability to correct temperature error due to local attenuation in any installation configuration. The conducted calibration provided an accurate temperature profile with a mean value of root mean square error (RMSE) of around 0.1 °C. At the same time, the mean of RMSE for the internally calibrated temperature was 0.3 °C. The absolute bias with respect to the reference thermometer was decreased from 0.27 °C for the DTS measurements to 0.08 °C for the PEST calibrated temperature. The absolute bias and RMSE for both the temperature data obtained from the DTS and the calibrated temperatures are shown in Figure 9.

**Figure 10:**
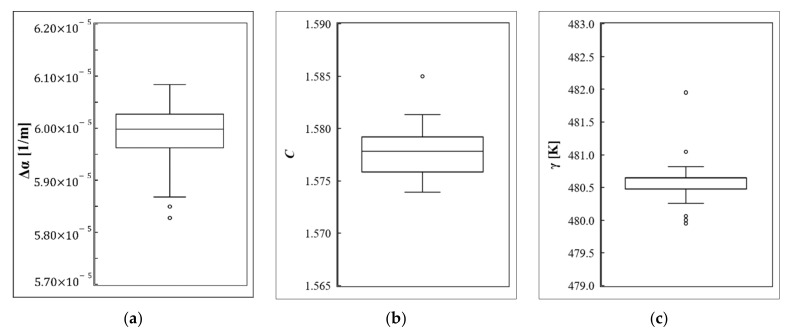
Estimated values of calibration parameters. (**a**) ∆α: differential attenuation parameter, (**b**) C: laser and DTS instrumentation parameter, and (**c**) γ : energy shift-related parameter.

In addition to the easier installation configuration, the current calibration technique provides some advantages related to the calibration process and parameter estimation. PEST can analyze the uncertainties and estimate the parameters in a complex model with large numbers of variables. Additionally, using the PEST tools, the sensitivity of the calibration model to the estimated parameters can be obtained. The calibration process by the PEST software tool is faster than the conventional solvers. Within the control file, the initial and boundary values for the parameter and the termination criteria can be defined, leading to more accurate and stable parameter estimation. The estimated values of calibration parameters during the two hours of measurement are shown in Figure 10. A tiny variation can be observed in all three estimated parameters, with only a few outlier values. This illustrates the fact that the measurement conditions and DTS characteristics remained constant during the two hours of measurement. 

**Figure 11:**
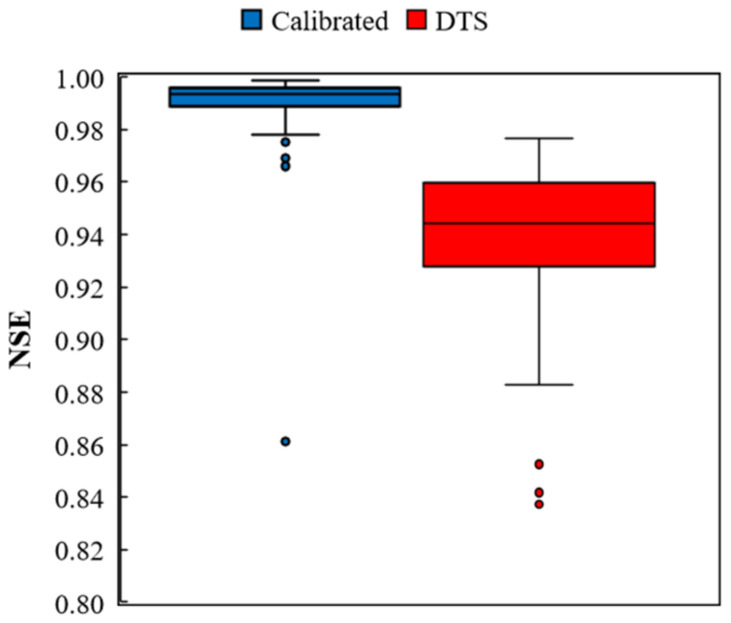
Nash–Sutcliffe Efficiency Index for calibrated and DTS temperature.

To assess the goodness of fit of the calibration model, the Nash–Sutcliff Efficiency Index (NSE) of the model was calculated by the PEST tools for all measurement time increments. The NSE index ranges from zero to one, while values near one suggest that the model has high predictive skill [30]. The plots of the NSE index for both the PEST calibration model and the DTS internal calibration model are presented in Figure 11. The outlier values under 0.8 are not presented in the figure. A calibration model with a high predictive ability was developed by the proposed calibration technique, compared to the internal calibration model. 

The improvements in the temperature measurement and the efficiency of the developed PEST calibration model are summarized in Table 1, in terms of the mean absolute bias (MAB), RMSE, and mean Nash–Sutcliff Efficiency Index (MNSE). 

The accuracy of temperature measurements was significantly improved by the calibration. The calibration’s reliability strongly depends on the accuracy of the thermometers. Considering the obtained absolute bias and the accuracy of the employed thermometers, the calibrated temperature measurements were obtained with the maximum possible error of ±0.52 °C. For the performed experiment, where the fiber was subjected to rapid temperature changes, the obtained accuracy was satisfactory. However, the same technique of calibration can be used with more accurate thermometers to improve the accuracy of temperature measurements.

## 4. Conclusions 

A fully dynamic calibration was developed using external thermometers to form the reference points. The real-time clocks of the DTS system and temperature sensors were precisely synchronized for the calibration. An experimental model was designed to calibrate the temperature data in sand subjected to water seepage. The calibration parameters were calculated using the PEST tools, which provided the best-fitted parameters in a short time. Application of the developed method is recommended based on the following conclusions obtained from this work:

- This approach eliminates the need for a reference bath with a constant temperature, which is a complicated task in many practical applications.
- This technique reduces the complexity of optical fiber installation for calibration intention. Small-sized thermometers can be placed at any location (even within the structure), together with a coil of optical fiber, to form reference sections/points.
- Using the PEST, the number of reference points can be increased for a better calibration, without a notable decrease in the speed and accuracy of the calibration.
- The suggested approach is appropriate for monitoring a structure subjected to rapid temperature variations.

Considering the conducted study, the following topics can be suggested as further works for the calibration of DTS data:

- A real-time thermometer with higher accuracy can be employed by this method to form a fully automatic calibration in real time. With the real-time calibrated measurement, any defect will be detected quickly and in the early stages, providing sufficient time for required intervention activities.
- The employment of the proposed calibration technique is recommended for future studies. This technique is particularly suitable for field measurements where the formation of constant temperature reference baths is more complex and costly.

## Figures and Tables

**Table 1:** Measurement improvement by the PEST calibration.

Index	DTS Measurement	PEST Calibrated
MAB	0.27 °C	0.08 °C
RMSE	0.30 °C	0.10 °C
MNSE	0.81	0.97

## Data Availability

The datasets analyzed during the current study are available on request from the corresponding author.
